# Author's reply

**DOI:** 10.1111/ina.12169

**Published:** 2015-04-24

**Authors:** S Agrawal, S Yamamoto

**Affiliations:** 1 South Asia Network for Chronic Disease, Public Health Foundation of IndiaNew Delhi, India; 2 Department of Non-Communicable Disease Epidemiology, London School of Hygiene and Tropical MedicineLondon, UK

Ragnar Rylander raises an interesting point about correcting for dietary magnesium in assessing preeclampsia/eclampsia risk. Magnesium is an essential mineral required for regulation of body temperature, nucleic acid, and protein synthesis and in maintaining nerve and muscle cell electrical potentials. Many women, especially those from disadvantaged backgrounds and in low- and middle-income countries, have low intakes of magnesium. Magnesium supplementation during pregnancy may be able to reduce fetal growth restriction and preeclampsia, and increase birth weight. Dietary magnesium thus been proven to be an important dietary element that has been related to hypertension, preeclampsia and eclampsia in research conducted in the West (Franz, 1987; Jain et al., 2010; James and Nelson-Piercy, 2004; Rylander, 2014). A study by Jain et al. (2010) showed that a reduction in the serum levels of calcium, magnesium, and zinc during pregnancy might be a possible contributor in the etiology of preeclampsia, and supplementation of these elements to diet may be of value to prevent preeclampsia. Another Indian study (Lambe et al., 2014) concluded that serum calcium, magnesium, and zinc can be considered as factors having a role in the etiopathogenesis of preeclampsia and as severity indicators in preeclamptic women.

However, a recent Cochrane review (Makrides et al., 2014) reports that there is not enough high-quality evidence to show that dietary magnesium supplementation during pregnancy is beneficial. Randomized and quasi-randomized trials assessing the effects of dietary magnesium supplementation during pregnancy were included in the review. In the analysis of all trials, oral magnesium supplementation compared with no magnesium was associated with no significant difference in preeclampsia risk (RR:0.87; 95%CI:0.58–1.32; three trials, 1042 women). Of the 10 trials included in the review, only two were judged to be of high quality overall. When an analysis was restricted to these two trials, none of the review's primary outcomes including preeclampsia were significantly different between the magnesium supplemented and control groups. A recent study (Gol Mohammadlou et al., 2008) involving Iranian women also found that the mean serum levels of calcium, magnesium, copper, and zinc between the preeclamptic patients in their third trimester of pregnancy and healthy normotensive pregnant women were not significant, which means that these trace elements might not be involved in the pathogenesis of preeclampsia.

Our study is limited by the non-availability of data on magnesium intake as NFHS-3 did not collect data on magnesium supplementation during pregnancy. But NFHS-3 has data on micronutrient intake during pregnancy such as iron and folic acid supplementation (for at least 90 days) and mother's consumption of various food items. We have tried redoing the analysis with the above potential predictors along with indoor air pollution. In the multivariable logistic regression analysis, even after controlling for iron and folic acid supplementation during pregnancy and dietary diversity (constructed variable, based on frequency of intake of specific food items), cooking with biomass and solid fuels still has a statistically significant effect (OR: 2.00; 95% CI: 1.17–3.42; *P* = 0.011) on the likelihood of symptoms suggestive of preeclampsia/eclampsia among Indian women. However, estimating the independent effect, we found that women consuming iron and folic acid supplementation during pregnancy (OR: 0.72; 95% CI: 0.53–0.99; *P* = 0.040) and women consuming an adequate diversified diet (OR: 0.76; 95% CI: 0.58–1.00; *P* = 0.049) had a lower likelihood of reporting preeclampsia/eclampsia symptoms than their counterparts.
